# Efficacy and safety of bevacizumab in patients with malignant melanoma: a systematic review and PRISMA-compliant meta-analysis of randomized controlled trials and non-comparative clinical studies

**DOI:** 10.3389/fphar.2023.1163805

**Published:** 2023-07-13

**Authors:** Xiao Han, Pu Ge, Siyu Liu, Dandan Yang, Jinzi Zhang, Xinpei Wang, Weiting Liang

**Affiliations:** ^1^ State Key Laboratory of Oncology in South China, Guangdong Key Laboratory of Nasopharyngeal Carcinoma Diagnosis and Therapy, Sun Yat-sen University Cancer Center, Guangzhou, China; ^2^ Department of Pharmacy, The Fifth Affiliated Hospital of Sun Yat-sat University, Zhuhai, China; ^3^ School of Traditional Chinese Medicine, Beijing University of Chinese Medicine, Beijing, China; ^4^ School of Stomatology, Shandong University, Jinan, China; ^5^ School of Pharmaceutical Sciences, Sun Yat-sen University, Guangzhou, China; ^6^ School of Humanities and Social Sciences, Harbin Medical University, Harbin, China; ^7^ Medical Equipment Department, Peking University First Hospital, Beijing, China

**Keywords:** melanoma, bevacizumab, angiogenesis inhibitors, meta-analysis, systematic review

## Abstract

**Background:** Malignant melanoma is a highly aggressive cancer that spreads and metastasizes quickly. In recent years, the antiangiogenic drug bevacizumab has been trialed to treat malignant melanoma. We conducted the first meta-analysis to examine the efficacy and safety of bevacizumab combined with other drugs in malignant melanoma.

**Methods:** We searched for randomized controlled trials (RCTs) and non-comparative clinical studies of bevacizumab combined with chemotherapy, targeted medicine, and interferon to treat malignant melanoma in PubMed, Embase, the Cochrane Library, and Web of Science. Meta-analysis of RCT was performed using Review Manager (version 5.4), and non-comparative meta-analysis was performed using R (version 4.0.3). The primary outcome was the objective response rate. Depending on the heterogeneity of the included studies, the pooled outcomes and 95% CI were calculated using either random-effects or fixed-effect models. Subgroup outcomes were calculated with possible relevant variables. Sensitivity analyses were carried out by excluding each study from the highly heterogeneous pooled results in turn. Funnel plot and Begg’s test were used to test the included studies' potential publication bias. The level of significance was set at *p* < 0.05.

**Results:** This meta-analysis included 20 trials: five RCTs and 15 non-comparative clinical studies with a total of 23 bevacizumab intervention arms. In 14 treatment arms, bevacizumab was combined with chemotherapy drugs such as fotemustine, dacarbazine, carboplatin/paclitaxel, and temozolomide. In six treatment arms, bevacizumab was combined with targeted medicines such as imatinib, everolimus, sorafenib, erlotinib, and temsirolimus. There were also six treatment arms that used bevacizumab in combination with interferon. The pooled objective response rate was 15.8% (95% CI, 11.4%–20.2%). Bevacizumab plus carboplatin/paclitaxel significantly increased the overall survival compared to carboplatin/paclitaxel (HR = 0.64, 95% CI, 0.49-0.85, *p* < 0.01). Fatigue, nausea, leukopenia, thrombocytopenia, and neutropenia were the most common adverse events. The pooled incidence of hypertension of all bevacizumab arms in malignant melanoma was 32.4% (95% CI, 24.5%–40.3%).

**Conclusion:** This study showed that bevacizumab combined with chemotherapy might be effective and well-tolerated in patients with stage III or IV unresectable malignant melanoma.

**Systematic Review Registration**: [https://www.crd.york.ac.uk/PROSPERO/display_record.php?RecordID=304625], identifier [CRD42022304625].

## 1 Introduction

Tumor cells exhibit high metabolic physiological characteristics due to their rapid growth and unlimited proliferation. Angiogenesis is a key regulator of solid tumor growth and metastasis. Vascular endothelial growth factor (VEGF) promotes tumor angiogenesis and can activate the VEGF signaling pathway by binding to tyrosine kinases on the VEGF receptor. When this pathway is activated, the endothelial cells will proliferate, solid tumor neovascularization will be promoted, and tumor growth will be accelerated (Garcia et al., 2020). In recent years, antiVEGF drug development has emerged as a hot topic of research in the field of antitumor targeted drugs.

Bevacizumab, a humanized monoclonal antibody, is the first antiangiogenic drug used in antitumor therapy. By binding VEGF-A, bevacizumab can block the interaction of VEGF-A with VEGF receptors, and the activation of the VEGF signaling pathway is thus inhibited. Researchers have conducted several studies *in vivo* and found that, on one hand, bevacizumab inhibits tumor cell growth through three mechanisms: inhibition of angiogenesis, induction of degeneration of newly formed blood vessels, and normalization of blood vessels. On the other hand, bevacizumab can also enhance the efficacy of cytotoxic drugs and immunotherapy, thus exerting an antitumor effect (Ferrara et al., 2004; Rahma and Hodi, 2019). Several clinical trials have shown that the combination of bevacizumab with chemotherapy or other targeted drugs can prolong the progression-free survival (PFS) and overall survival (OS) of patients with solid tumors such as metastatic colorectal cancer (mCRC), non-small cell lung cancer (NSCLC), and breast cancer (Hurwitz et al., 2004; Sandler et al., 2006; Miller et al., 2007; Rini et al., 2010; Lai et al., 2011; Perren et al., 2011; Kelly et al., 2012). The specific antiangiogenic mechanism of bevacizumab makes it essential in the therapeutic regimen of angiogenesis-driven solid tumors.

Malignant melanoma is a type of cancer with a high level of aggressiveness, rapid progression, and metastasis. The incidence of malignant melanoma is increasing worldwide (CSCO Melanoma Expert Committee, 2012). Its prevalence in China is increasing at an annual rate of 3%–5% (Guo et al., 2015). Malignant melanoma is a serious threat to the life and health of people. Previously, the treatment options for melanoma were relatively homogeneous. Chemotherapy was the primary treatment method for unresectable advanced melanoma. However, chemotherapy alone had limited therapeutic benefits. Antiangiogenic drugs, which inhibit solid tumor angiogenesis, can have antitumor effects and are used to treat a variety of solid tumors, including malignant melanoma. Clinical trials have shown that combining recombinant human vascular endothelial inhibitor injection with dacarbazine is more effective than administering dacarbazine alone in the first-line treatment of patients with metastatic melanoma, improving PFS and OS (Cui et al., 2013). Basic and clinical studies have also been conducted to investigate the feasibility of combining bevacizumab with other drugs to treat malignant melanoma. The effectiveness and safety of bevacizumab in the treatment of malignant melanoma have not yet been examined in a systematic review or meta-analysis. Hence, we performed a systematic review to investigate the effectiveness and safety of bevacizumab-containing therapies in patients with malignant melanoma.

## 2 Materials and methods

This study followed the Preferred Reporting Items for Systematic Reviews and Meta-Analyses (PRISMA) guidelines (See Supplementary Table S1 for details). Before the formal start of this study, it was registered on the PROSPERO platform, with the registration number CRD42022304625.

### 2.1 Inclusion and exclusion criteria

#### 2.1.1 Inclusion criteria

1) Adult patients with a pathological diagnosis of malignant melanoma, regardless of gender, race, tumor stage, and tumor position. 2) Interventions in the experimental group must include bevacizumab treatment, with unlimited types and amounts of antitumor drugs in combination and treatment sublines at the time of the subject’s participation in the study, and unlimited treatment regimens in the control group. Prospective clinical trials that did not contain a control group were also included in the study. 3) Included studies were required to report at least one of the following outcome indicators: effectiveness indicators including objective response rate (ORR), disease control rate (DCR), number of people in complete remission (CR), number of people in partial remission (PR), number of people with stable disease (SD), PFS, and OS. Safety indicators included the number of all types of adverse events (AEs) and grade III/IV AEs. 4) The type of study included should be RCTs or non-randomized controlled prospective clinical studies.

#### 2.1.2 Exclusion criteria

1) Article types were abstracts, reviews, case reports, and case series. 2) Study types were cellular or animal studies and phase I clinical studies. 3) For papers that repeatedly reported the results of the same study, only the one with complete results was included for data analysis. 4) The full text of the paper was not available. 5) Papers that were not written in English.

### 2.2 Data source and search strategy

The literature search was conducted by three researchers in the databases PubMed, Embase, Web of Science, and the Cochrane Library. In addition, the researchers searched clinical studies registered in the clinical trials website. Studies published up to 10 January 2022 were searched with the following search terms: (bevacizumab* OR Mvasi OR Avastin) AND (Melanoma OR melanoma). The search strategy for each database is shown in Supplementary Table S2. To avoid missing relevant studies, we checked the reference sections of the original studies and the reviews that were retrieved. Following the completion of the search, the researchers imported all of the retrieved literature into EndNote X9 software and removed any duplicates.

Two researchers independently read the titles and abstracts of the literature during the initial screening phase and excluded irrelevant literature based on the inclusion and exclusion criteria, and two researchers read the full text of eligible articles and finalized the studies for inclusion in the systematic review and meta-analysis during the re-screening phase. Both researchers discussed the included and excluded literature at the end of the initial and re-screening phases of the literature. In the event of a disagreement, a third investigator was involved in to help reach a consensus.

### 2.3 Quality assessment

RCTs were assessed using the Cochrane risk of bias tool, provided complete outcome data, and reported no selective outcome without other bias (Higgins et al., 2011). Non-randomized controlled prospective clinical studies were assessed by the methodological index for non-randomized studies (MINORS) (Slim et al., 2003). Because there was no controlled group in non-randomized controlled prospective clinical studies, these items were not evaluated: adequate control group, contemporary groups, baseline equivalence of groups, and adequate statistical analyses.

### 2.4 Data extraction

Two researchers extracted data independently using pre-designed data extraction forms, and any disagreements were resolved with a joint discussion with the third author. The following data were extracted from each study: the first author’s name, year of publication, study design, median follow-up time, sample size, median age, sex of patients, ECOG scoring range of patients, other kinds of therapeutic drugs used, clinical trial line, and main outcomes. The primary outcome was the ORR according to the evaluation of the researchers. The following data were also extracted if the study contained: DCR, OS, PFS, and number of AEs. ORR was defined as the percentage of patients with measurable disease who achieved CR or PR. DCR was defined as the percentage of patients with measurable disease who achieved CR, PR, or SD. PFS was defined as the time from the initiation of bevacizumab to disease progression, death from any cause, or last follow-up. OS was defined as the time from initiation of bevacizumab to death from any cause or last follow-up. AE was defined as any adverse or unintended sign, symptom, or disease caused by a drug. Although the raw survival data were hardly accessed, the extracted data including the number of patients on PFS and OS at 3, 6, 9, and 12 months from the Kaplan–Meier curves (K-M curves) were obtained by Engauge Digitizer version 11.1 software.

### 2.5 Data analysis

Meta-analysis of RCT was performed using Review Manager (version 5.4), and non-comparative single-arm meta-analysis was performed using R (version 4.0.3). In the non-comparative single-arm meta-analysis, the raw data that did not conform to the normal distribution were transformed using appropriate data transformation methods (inverse sine transformation, Freeman–Tukey double inverse sine transformation, log transformation, and logit transformation). Data were analyzed using either a random-effects model or a fixed-effects model based on the results of the heterogeneity test, and the combined results were expressed by 95% CI (with upper and lower limits). Between-study heterogeneity was assessed using Cochran’s Q test and I^2^. For the Q test, a *p*-value less than 0.05 indicated significant heterogeneity. For the I^2^ , an I^2^ value greater than 50% indicated significant heterogeneity. When the combined results showed low heterogeneity, a fixed-effects model was used for analysis; otherwise, a random-effects model was used for analysis. Factors contributing to the risk of bias were identified by subgroup analysis, and subgroup outcomes were calculated for ORR, DCR, PFS rate and OS rate at 3, 6, 9, and 12 months, with possible relevant variables (combined with chemotherapy, combined with targeted agents, combined with interferon, disease stage, treatment line, study type, and pathology type). Sensitivity analyses were performed by sequentially excluding each study from the combined outcomes. Potential publication bias was analyzed using funnel plots and Begg’s test. Unless otherwise stated, *p* < 0.05 was considered statistically significant.

## 3 Results

### 3.1 Study selection

**FIGURE 1:**
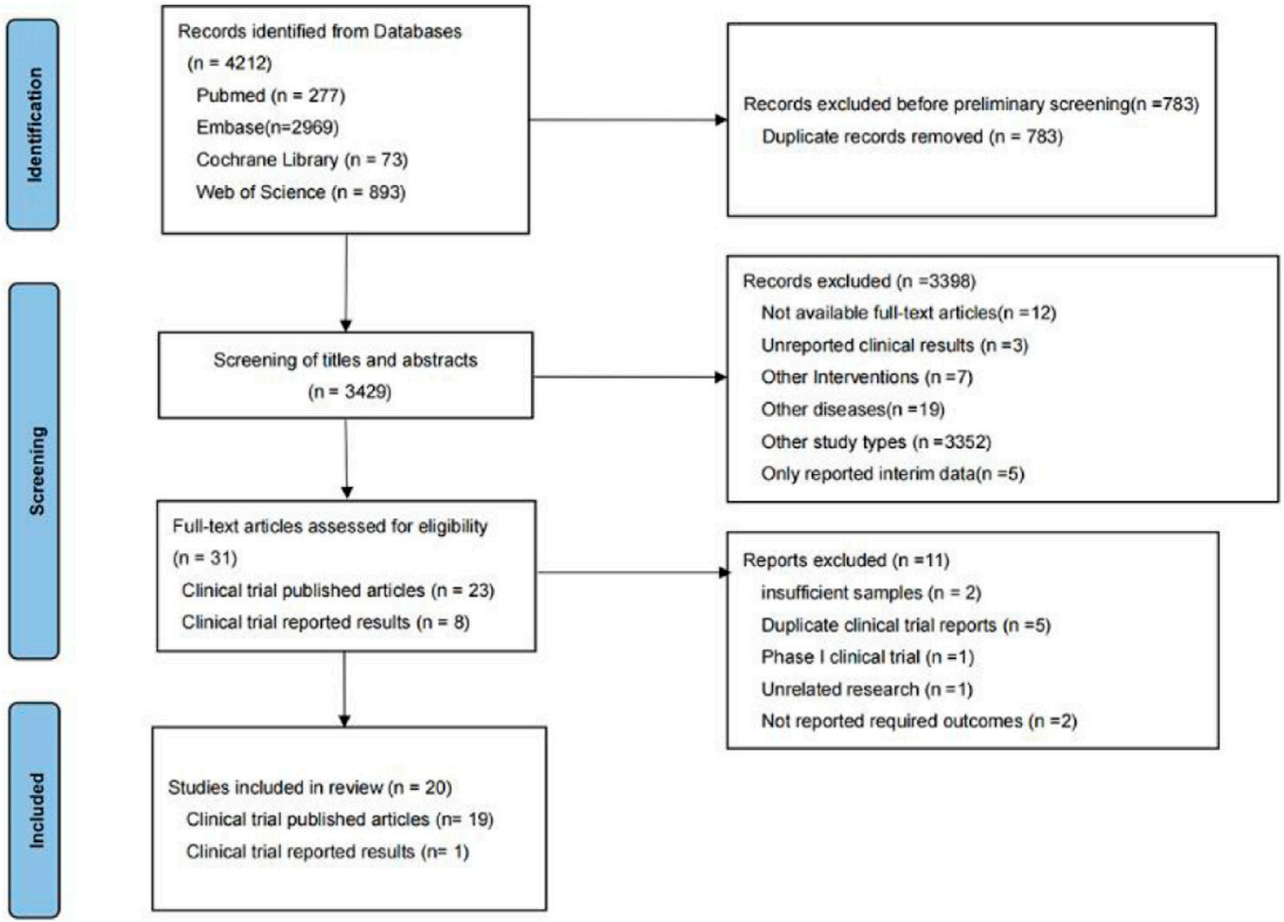
Flowchart of study selection for meta-analysis.

In the preliminary search, 4,212 relevant studies were retrieved, i.e., 277 studies from PubMed, 2,969 studies from Embase, 73 studies from Cochrane Library, and 893 studies from Web of Science. In total, 783 duplicate studies were removed. The remaining 3,429 studies were included in the initial screening process for this study, while initial screening was performed by reading the titles and abstracts of the studies. A total of 3,398 studies were excluded during the initial screening process, leaving 31 studies included in the re-screening phase of this study. In the re-screening phase of this study, 11 of the 31 studies were excluded. Finally, 20 studies were screened for systematic review and meta-analysis. The reasons why studies were excluded during the initial and re-screening stages can be seen in Figure 1.

### 3.2 Basic characteristics of the included studies

The 20 included studies included five RCTs (U.S.National Library of Medicine, 2018; Kevin et al., 2012; McWilliams et al., 2018; Varker et al., 2007; Yan et al., 2021) and 15 non-randomized controlled prospective clinical studies (Perez et al., 2009; Del Vecchio et al., 2010; Hainsworth et al., 2010; Vihinen et al., 2010; Grignol et al., 2011; Schuster et al., 2012; Von Moos et al., 2012; Kottschade et al., 2013; Slingluff et al., 2013; Mahalingam et al., 2014; Ferrucci et al., 2015; Flaherty et al., 2015; Spitler et al., 2015; Mudigonda et al., 2016; Piperno-Neumann et al., 2016). A total of 23 bevacizumab treatment arms were included in all studies. In 14 treatment arms, bevacizumab was combined with chemotherapy drugs such as fotemustine, dacarbazine, carboplatin/paclitaxel, and temozolomide. In six treatment arms, bevacizumab was combined with targeted medicines such as imatinib, everolimus, sorafenib, erlotinib, and temsirolimus. There were also six treatment arms that used bevacizumab in combination with interferon. All studies contained 1,095 patients with unresectable, stage III or stage IV malignant melanoma. The age range of the patients was 22–89 (unit: year), and 61.2% were male. The basic characteristics of all studies can be seen in Table 1.

**TABLE 1:** Characteristics of studies in qualitative synthesis.

Research		Study design	Object	Intervention	Sample size	Median age (year, range)	Gender (male/female)	Treatment line	ECOG	Median follow-up years, month	Outcome
2010	Del Vecchio, M.	Non-comparative	Metastatic nonchoroidal melanoma	Bevacizumab/fotemustine	20	54 (22–75)	12/8	First-line	0	NR	ORR, DCR, OS, and AE
2014	Ferrucci, P. F.	Non-comparative	Metastatic melanoma	Bevacizumab/dacarbazine	40	56 (31–78)	24/13	Unreported	0–1	NR	ORR, DCR, PFS, OS, and AE
2015	Flaherty, K. T.	Non-comparative	Metastatic melanoma	Bevacizumab/imatinib	23	63 (49–86)	16/7	Unreported	0–1	NR	ORR, DCR, PFS, and AE
2011	Grignol, V. P.	Non-comparative	Metastatic melanoma	Bevacizumab/high-dose IFN-α	25	56 (31–79)	16/9	Unreported	0–1	NR	ORR, DCR, OS, PFS, and AE
2010	Hainsworth, J. D.	Non-comparative	Metastatic melanoma	Bevacizumab/everolimus	57	70 (36–88)	39/18	Unreported	0–2	13	ORR, DCR, OS, PFS, and AE
2012	Kim, K. B.	RCT	Metastatic melanoma not of uveal origin	Carboplatin/paclitaxel	71	60 (28–83)	50/21	First-line	0–1	13.3	ORR, OS, PFS, and AE
				Bevacizumab/carboplatin/paclitaxel	143	60 (27–85)	98/45	First-line	0–1	13	ORR, OS, PFS, and AE
2013	Kottschade, L. A.	Randomized, non-comparative	Metastatic melanoma	Bevacizumab/emozolomide	42	57 (25–82)	24/18	Unreported	0–1	16	ORR, OS, PFS, and AE
				Bevacizumab/carboplatin/nab-paclitaxel	51	57 (22–83)	29/22	Unreported	0–1	16.5	ORR, OS, PFS, and AE
2014	Mahalingam, D.	Non-comparative	Metastatic melanoma	Bevacizumab/sorafenib	14	61 (43–77)	9/5	Unreported	0–1	NR	ORR, PFS, and AE
2018	McWilliams, R. R.	RCT	Metastatic melanoma	Carboplatin/paclitaxel/bevacizumab	75	59	54/21	Unreported	0–1	38.5	ORR, OS, PFS, and AE
				Carboplatin/paclitaxel/bevacizumab/everolimus	73	58	40/33	Unreported	0–1	38.5	ORR, OS, PFS, and AE
2016	Mudigonda, T. V.	Non-comparative	Metastatic melanoma	Bevacizumab/erlotinib	28	NR	17/11	Unreported	0–1	NR	ORR, OS, PFS, and AE
2009	Perez, D. G.	Non-comparative	Metastatic melanoma	Bevacizumab/carboplatin/paclitaxel	53	55 (30–84)	33/20	Unreported	0–2	NR	ORR, DCR, OS, PFS, and AE
2016	Piperno-Neumann, S.	Non-comparative	Metastatic uveal melanoma	Bevacizumab/temozolomide	35	55 (29–72)	19/16	First-line	0–1	26	ORR, OS, PFS, and AE
2012	Schuster, C.	Non-comparative	Metastatic melanoma	Bevacizumab	35	63 (26–77)	19/16	Unreported	0–1	NR	ORR, DCR, OS, PFS, and AE
2013	Slingluff Jr, C. L.	Non-comparative	Unresectable stage III to IV melanoma	Bevacizumab/temsirolimus	17	65 (23–81)	11/6	unreported	0–1	NR	ORR, DCR, and AE
2015	Spitler, L. E.	Non-comparative	Unresectable stage III to IV melanoma	Bevacizumab/nab-paclitaxel	50	62 (25–89)	32/18	First-line	0–1	41.6	ORR, DCR, OS, PFS, and AE
2007	Varker, K. A.	RCT	Metastatic melanoma	Bevacizumab/low-dose Interferon *α*-2b	16	64 (28–74)	9/7	Unreported	0–1	NR	ORR, DCR, OS, PFS, and AE
				Bevacizumab	16	54 (39–83)	9/7	Unreported	0–1	NR	ORR, DCR, OS, PFS, and AE
2010	Vihinen, P. P.	Non-comparative	Metastatic melanoma	Bevacizumab/dacarbazine/iFN-a2a	26	53 (31–69)	19/7	First-line	0–1	10	ORR, DCR, OS, PFS, and AE
2012	Von Moos, R.	Non-comparative	Metastatic melanoma	Bevacizumab/temozolomide	62	59 (29–82)	40/22	First-line	0–2	20.1	ORR, DCR, OS, PFS, and AE
2021	Yan, X.	RCT	Unresectable stage III to IV melanoma	Carboplatin/paclitaxel	38	60 (32–74)	17/21	First-line	0–1	44.5	ORR, DCR, OS, PFS, and AE
				Bevacizumab/carboplatin/paclitaxel	76	57.5 (29–73)	26/50	First-line	0–1	45.2	ORR, DCR, OS, PFS, and AE
2020	NCT02158520*	RCT	Metastatic melanoma	Bevacizumab/nab-paclitaxel	12	60 (37–77)	8/4	First-line	0–1	NR	ORR, OS, PFS, and AE
				Ipilimumab	12	61 (38–81)	7/4	First-line	0–1	NR	ORR, OS, PFS, and AE

NR, not reported; ORR, objective response rate; DCR, disease control rate; PFS, Progression-free Survival; OS, overall survival; AE, adverse event; *: From the clinical trials website. Control information is incomplete, so only the bevacizumab group was included in the analysis.

### 3.3 Risk of bias (ROB) assessment

**FIGURE 2:**
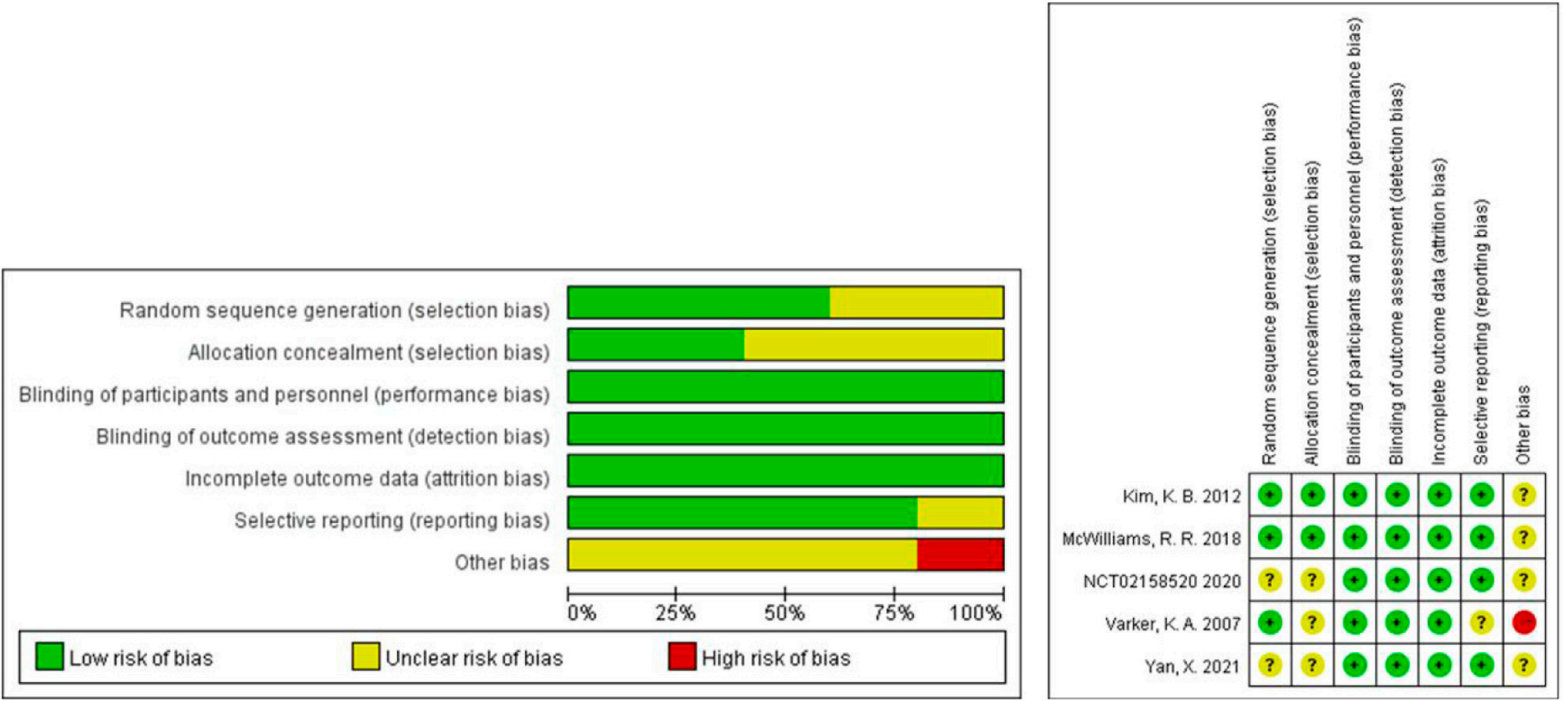
Risk of bias evaluation of RCT.

The Cochrane tool was used to evaluate the risk of bias of the five included RCTs (U.S.National Library of Medicine, 2018; Kevin et al., 2012; McWilliams et al., 2018; Varker et al., 2007; Yan et al., 2021). Because of the small sample size and open label, one study was more likely to have other bias (Varker et al., 2007). No RCTs were excluded from the meta-analysis due to excessive risk of bias. The risk of bias in the other 15 non-randomized controlled prospective clinical studies was assessed using MINORS (Perez et al., 2009; Del Vecchio et al., 2010; Hainsworth et al., 2010; Vihinen et al., 2010; Grignol et al., 2011; Schuster et al., 2012; Von Moos et al., 2012; Kottschade et al., 2013; Slingluff et al., 2013; Mahalingam et al., 2014; Ferrucci et al., 2015; Flaherty et al., 2015; Spitler et al., 2015; Mudigonda et al., 2016; Piperno-Neumann et al., 2016). The 15 studies received ratings ranging from 8 to 16. No non-randomized controlled prospective clinical studies were excluded from the meta-analysis due to excessive risk of bias. Figure 2 and Table 2 show the risk of bias evaluation results.

**TABLE 2:** Risk of bias evaluation of non-randomized controlled prospective clinical trials.

Research	I	II	III	IV	V	VI	VII	VIII	Scores
Del Vecchio, M. 2010 (Del Vecchio et al., 2010)	2	1	2	2	0	2	2	2	13
Ferrucci, P. F. 2014 (Ferrucci et al., 2015)	2	1	2	2	0	2	2	2	13
Flaherty, K. T. 2015 (Flaherty et al., 2015)	2	1	2	2	0	2	2	1	12
Grignol, V. P. 2011 (Grignol et al., 2011)	2	1	2	2	0	2	2	1	12
Hainsworth, J. D. 2010 (Hainsworth et al., 2010)	2	1	2	2	0	2	2	2	13
Kottschade, L. A. 2013 (Kottschade et al., 2013)	2	2	2	2	0	2	2	1	13
Mahalingam, D. 2014 (Mahalingam et al., 2014)	2	2	1	2	0	2	2	2	13
Mudigonda, T. V. 2016 (Mudigonda et al., 2016)	2	1	1	1	0	2	1	0	8
Perez, D. G. 2009 (Perez et al., 2009)	2	2	2	2	0	2	2	2	14
Piperno-Neumann, S. 2016 (Piperno-Neumann et al., 2016)	2	1	2	2	0	2	2	2	13
Schuster, C. 2012 (Schuster et al., 2012)	2	2	2	2	0	2	2	2	14
Slingluff Jr, C. L. 2013 (Slingluff et al., 2013)	2	2	1	2	0	1	2	1	11
Spitler, L. E. 2015 (Spitler et al., 2015)	2	2	2	2	0	2	2	0	12
Vihinen, P. P. 2010 (Vihinen et al., 2010)	2	1	2	2	0	2	2	0	11
Von Moos, R. 2012 (Von Moos et al., 2012)	2	2	2	2	2	2	2	2	16

I, a stated aim of the study; II, inclusion of consecutive patients; III, prospective collection of data; IV, endpoint appropriate to the study aim; V, unbiased evaluation of endpoints; VI, follow-up period appropriate to the major endpoint; VII, loss to follow-up not exceeding 5%; VIII, prospective calculation of the sample size.

### 3.4 Results of non-comparative single-arm meta-analysis in the bevacizumab intervention group

#### 3.4.1 Efficacy

##### 3.4.1.1 Objective response rate (ORR)

**FIGURE 3:**
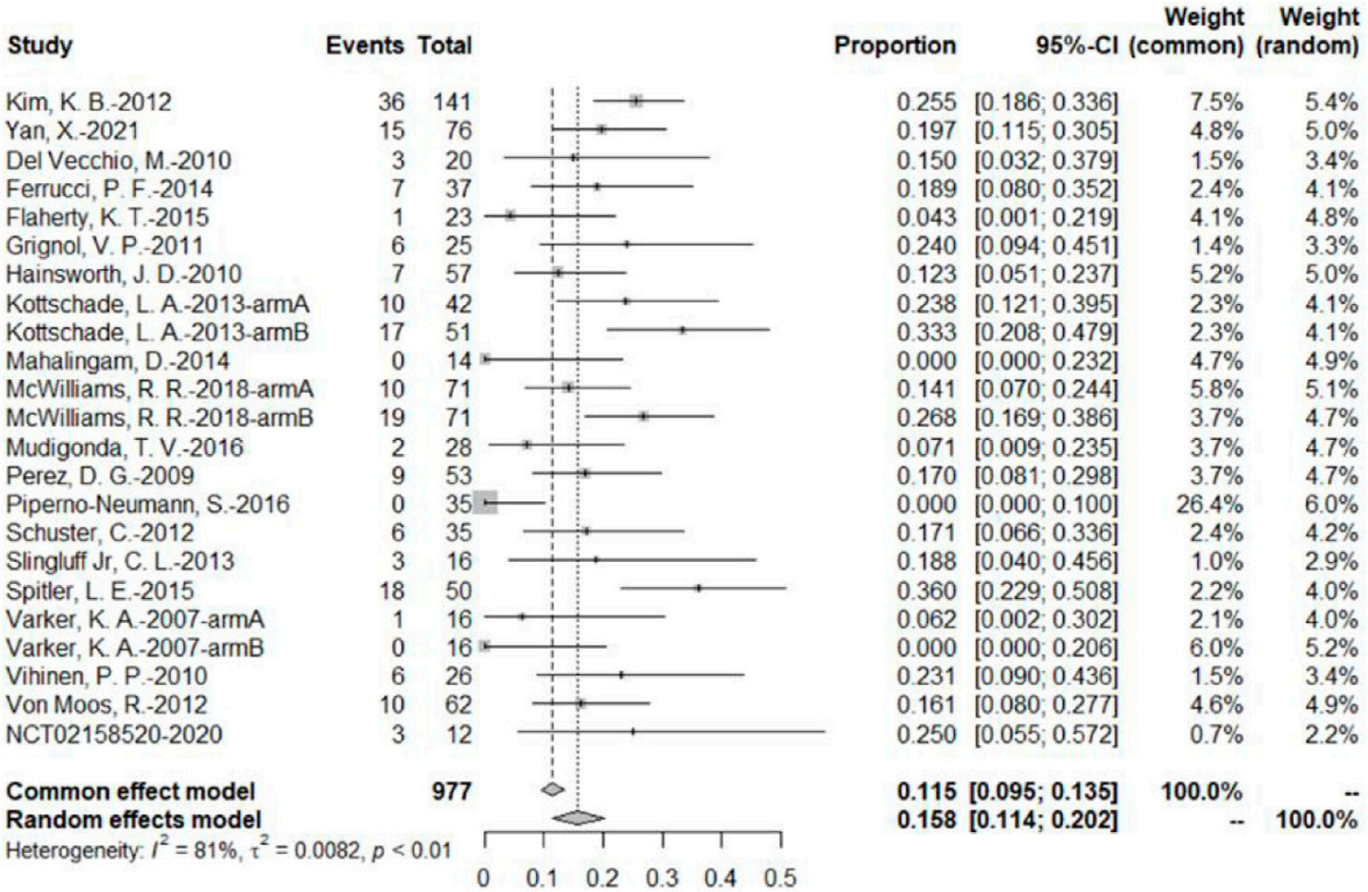
ORR of bevacizumab in malignant melanoma based on the bevacizumab group.

The 20 studies included a total of 23 bevacizumab intervention arms, and the ORR was reported in all 23 bevacizumab intervention arms (U.S.National Library of Medicine, 2018; Kevin et al., 2012; McWilliams et al., 2018; Varker et al., 2007; Yan et al., 2021; Del Vecchio et al., 2010; Ferrucci et al., 2015; Flaherty et al., 2015; Grignol et al., 2011; Hainsworth et al., 2010; Kottschade et al., 2013; Mahalingam et al., 2014; Mudigonda et al., 2016; Perez et al., 2009; Piperno-Neumann et al., 2016; Schuster et al., 2012; Slingluff et al., 2013; Spitler et al., 2015; Vihinen et al., 2010; Von Moos et al., 2012). Because of the relatively high heterogeneity, the results were combined using a random-effects model. The original rate was used to combine the data. In the bevacizumab intervention group, the pooled ORR was 15.8% (95% CI, 11.4%–20.2%, *I* ^*2*^ = 81%, and *p* < 0.01) (Figure 3).

##### 3.4.1.2 Disease control rate (DCR)

**FIGURE 4:**
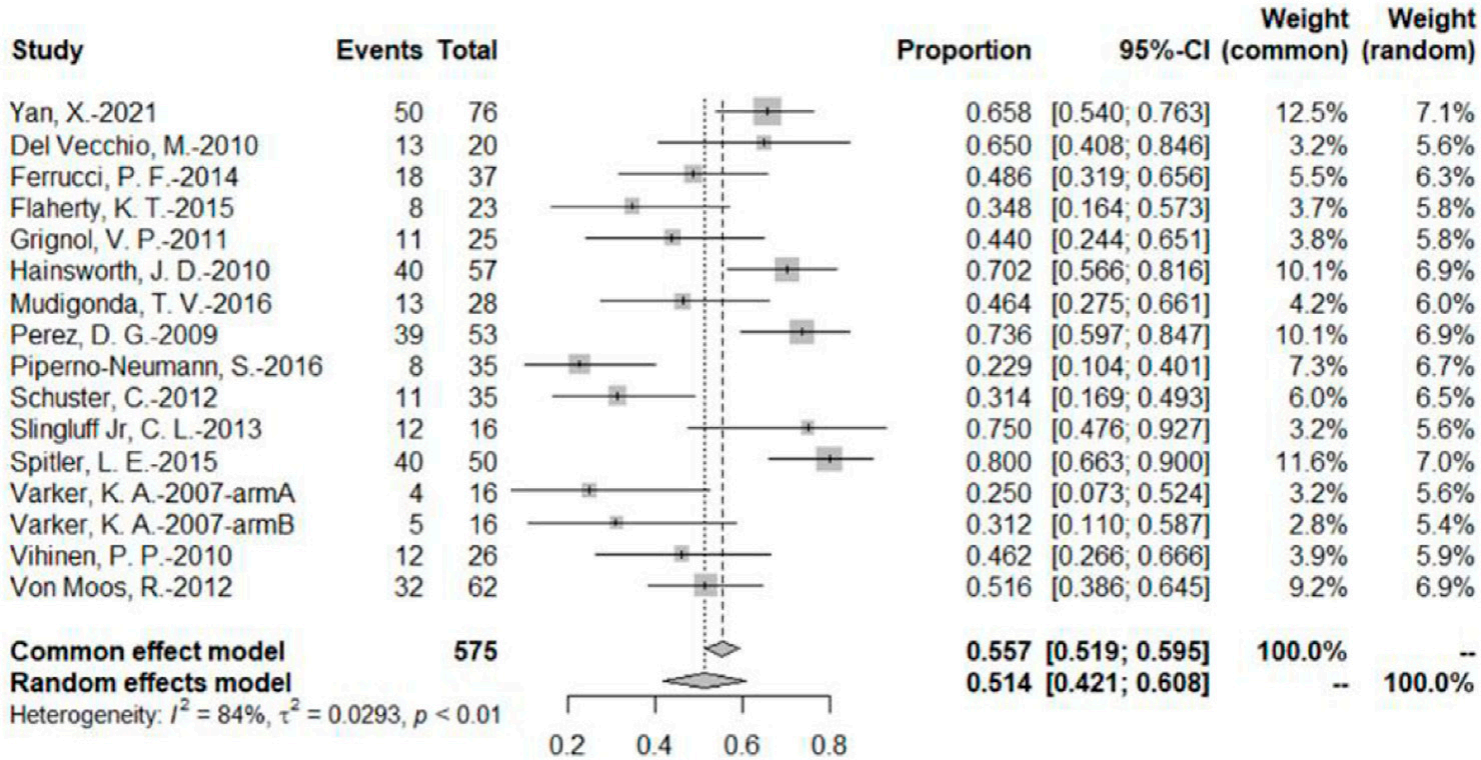
DCR of bevacizumab in malignant melanoma based on the bevacizumab group.

The DCR was reported by 16 bevacizumab intervention arms (Varker et al., 2007; Perez et al., 2009; Del Vecchio et al., 2010; Hainsworth et al., 2010; Vihinen et al., 2010; Grignol et al., 2011; Schuster et al., 2012; Von Moos et al., 2012; Slingluff et al., 2013; Ferrucci et al., 2015; Flaherty et al., 2015; Spitler et al., 2015; Mudigonda et al., 2016; Piperno-Neumann et al., 2016; Yan et al., 2021). Results were combined using a random-effects model due to relatively high heterogeneity. The original rate was used to combine the data. The combined DCR was 51.4% (95% CI, 42.1%–60.8%, *I* ^2^ = 84%, and *p* < 0.01) (Figure 4).

##### 3.4.1.3 Progression-free survival rate at different time points

**FIGURE 5:**
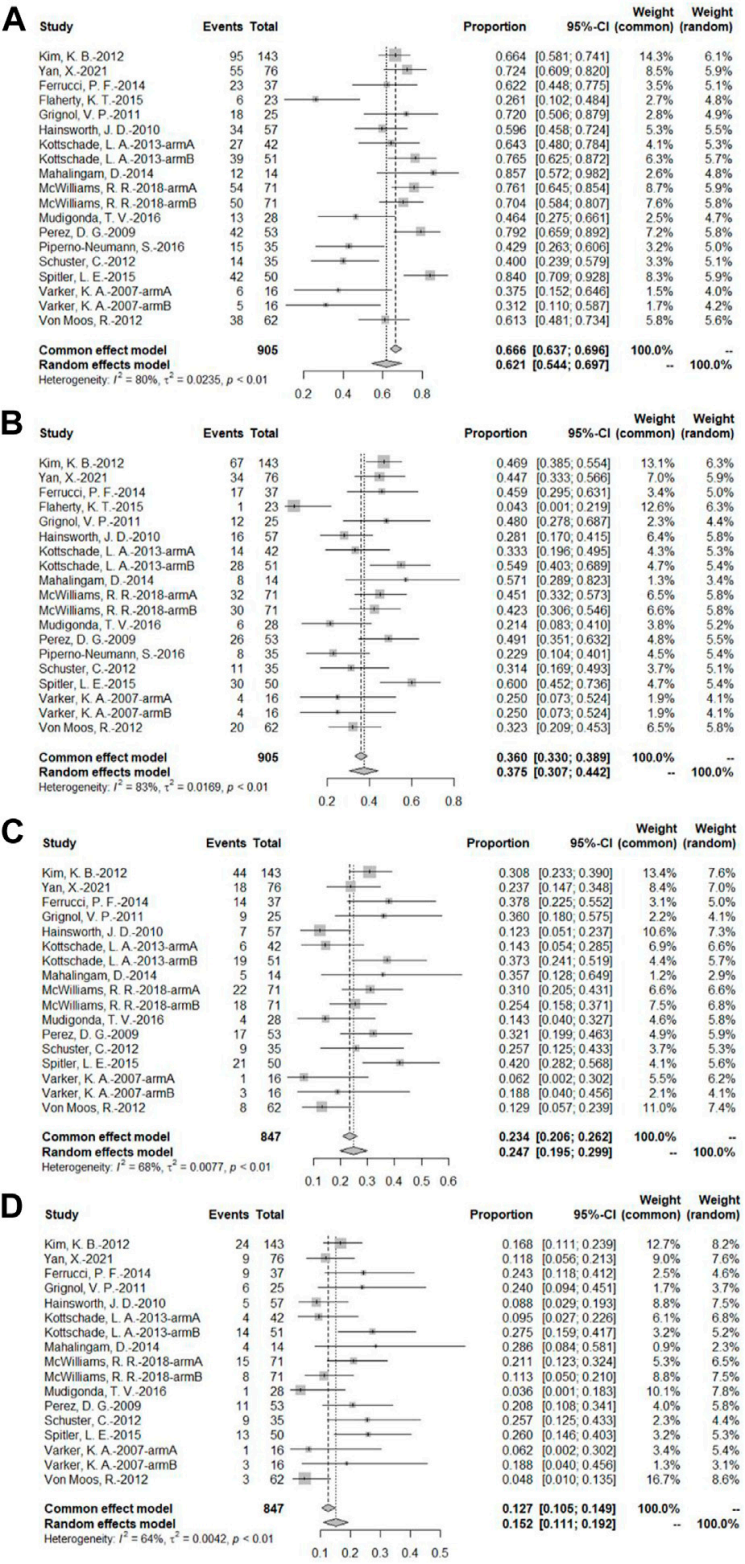
**(A)** PFS at 3 months based on the bevacizumab group; **(B)** PFS at 6 months based on the bevacizumab group; **(C)** PFS at 9 months based on the bevacizumab group; **(D)** PFS at 12 months based on the bevacizumab group.

A total of 16 bevacizumab intervention arms reported PFS (Varker et al., 2007; Perez et al., 2009; Hainsworth et al., 2010; Grignol et al., 2011; Kevin et al., 2012; Schuster et al., 2012; Von Moos et al., 2012; Kottschade et al., 2013; Mahalingam et al., 2014; Ferrucci et al., 2015; Flaherty et al., 2015; Spitler et al., 2015; Mudigonda et al., 2016; Piperno-Neumann et al., 2016; McWilliams et al., 2018; Yan et al., 2021), with 14 providing a Kaplan–Meier curve of PFS (Varker et al., 2007; Perez et al., 2009; Hainsworth et al., 2010; Grignol et al., 2011; Kevin et al., 2012; Schuster et al., 2012; Von Moos et al., 2012; Kottschade et al., 2013; Mahalingam et al., 2014; Ferrucci et al., 2015; Spitler et al., 2015; Mudigonda et al., 2016; McWilliams et al., 2018; Yan et al., 2021). Because of the relatively high heterogeneity, the results were combined using a random-effects model. To combine the data, the original rate was used. The PFS rates at 3, 6, 9, and 12 months were 62.1% (95% CI, 54.4%–69.7%, *I* ^2^ = 80%, and *p* < 0.01), 37.5% (95% CI, 30.7%–44.2%, *I* ^2^ = 83%, and *p* < 0.01), 24.7% (95% CI, 19.5%–29.9%, *I* ^2^ = 68%, and *p* < 0.01), and 15.2% (95% CI, 11.1%–19.2%, *I* ^2^ = 64%, and *p* < 0.01), respectively (Figure 5).

##### 3.4.1.4 Overall survival rate at different time points

**FIGURE 6:**
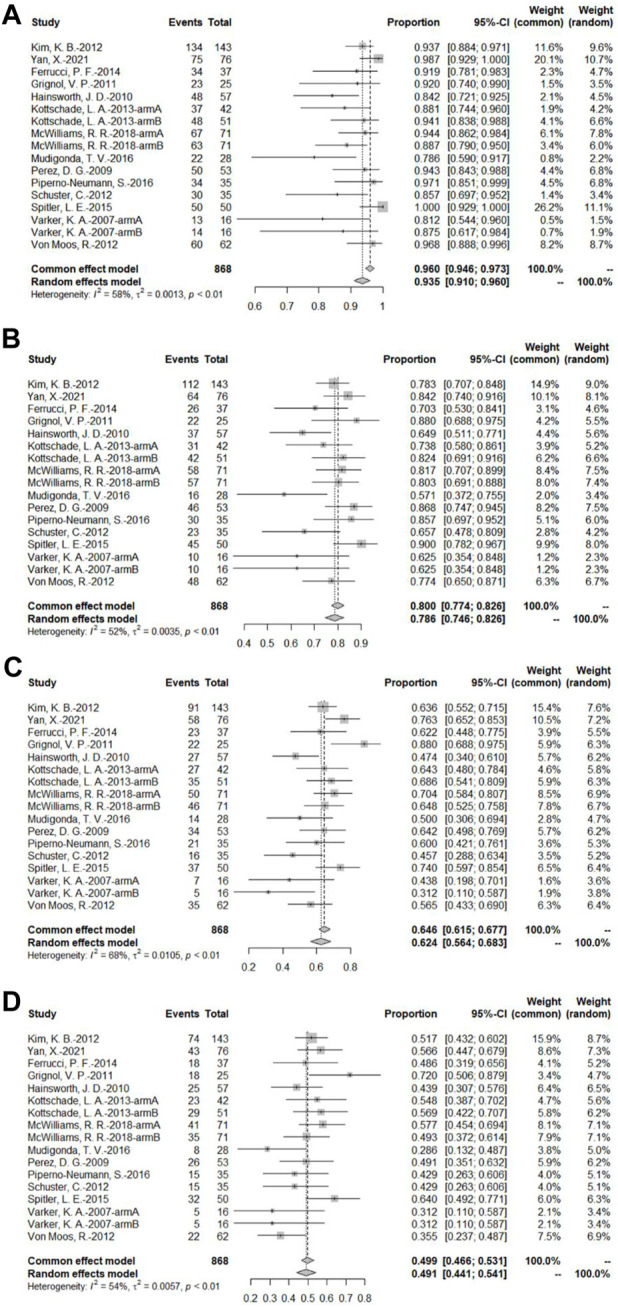
**(A)** OS at 3 months based on the bevacizumab group; **(B)** OS at 6 months based on the bevacizumab group; **(C)** OS at 9 months based on the bevacizumab group; **(D)** OS at 12 months based on the bevacizumab group.

A total of 14 bevacizumab intervention arms reported OS and provided a Kaplan–Meier curve of OS (Varker et al., 2007; Perez et al., 2009; Hainsworth et al., 2010; Grignol et al., 2011; Kevin et al., 2012; Schuster et al., 2012; Von Moos et al., 2012; Kottschade et al., 2013; Ferrucci et al., 2015; Spitler et al., 2015; Mudigonda et al., 2016; Piperno-Neumann et al., 2016; McWilliams et al., 2018; Yan et al., 2021). Results were combined using a random-effects model due to relatively high heterogeneity. The original rate was used to combine the data. The OS rates of 3, 6, 9, and 12 months were 93.5% (95% CI, 91.5%–96.0%, *I* ^2^ = 58%, and *p* < 0.01), 78.6% (95% CI, 74.6%–82.6%, *I* ^2^ = 52%, and *p* < 0.01), 62.4% (95% CI, 56.4%–68.3%, *I* ^2^ = 68%, and *p* < 0.01), and 49.1% (95% CI, 44.1%–54.1%, *I* ^2^ = 54%, *p* < 0.01), respectively (Figure 6).

#### 3.4.2 Safety

We combined data from the included studies on total adverse events and rates of each type of adverse event. In five studies, seven bevacizumab intervention arms reported the number of adverse events of all grades (Del Vecchio et al., 2010; Kevin et al., 2012; Ferrucci et al., 2015; Spitler et al., 2015; Yan et al., 2021). The rate of adverse events across all grades was 98.6% (95% CI, 97.1%–100%, *I* ^2^ = 0%, and *p* = 0.42). The number of grade III/IV adverse events was reported by 11 bevacizumab intervention arms in nine studies (U.S.National Library of Medicine, 2018; Kevin et al., 2012; McWilliams et al., 2018; Varker et al., 2007; Yan et al., 2021; Del Vecchio et al., 2010; Ferrucci et al., 2015; Spitler et al., 2015; Von Moos et al., 2012). The rate of grade III/IV adverse events was 58.0% (95% CI, 43.7%–72.3%, *I* ^2^ = 95%, and *p* 0.01). The five most common adverse events were fatigue, nausea, leukopenia, thrombocytopenia, and neutropenia, and the five most common grade III/IV adverse events were neutropenia, leukopenia, peripheral neuropathy, thrombocytopenia, and anemia.

We identified four adverse events from all types of adverse events that were more likely to be related to antiangiogenesis: hypertension, proteinuria, hemorrhage, and thrombosis.

There were 21 bevacizumab intervention arms that reported hypertension rates (U.S.National Library of Medicine, 2018; Kevin et al., 2012; McWilliams et al., 2018; Varker et al., 2007; Yan et al., 2021; Del Vecchio et al., 2010; Flaherty et al., 2015; Grignol et al., 2011; Hainsworth et al., 2010; Kottschade et al., 2013; Mahalingam et al., 2014; Mudigonda et al., 2016; Perez et al., 2009; Piperno-Neumann et al., 2016; Schuster et al., 2012; Slingluff et al., 2013; Spitler et al., 2015; Vihinen et al., 2010). When the data were combined using the original rates, the overall rate of hypertension was 32.4% (95% CI, 24.5%–40.3%, *I* ^2^ = 86%, and *p* < 0.01).

Proteinuria was reported in 15 of the bevacizumab intervention arms (U.S. National Library of Medicine, 2018; McWilliams et al., 2018; Varker et al., 2007; Yan et al., 2021; Ferrucci et al., 2015; Flaherty et al., 2015; Grignol et al., 2011; Hainsworth et al., 2010; Mahalingam et al., 2014; Piperno-Neumann et al., 2016; Schuster et al., 2012; Slingluff et al., 2013; Vihinen et al., 2010). The original rate was used to determine the overall rate of proteinuria: 26.3% (95% CI, 17.3%–35.2%, *I* ^2^ = 81%, and *p* < 0.01). Due to lack of data, grade III/IV hypertension and proteinuria rates were not calculated.

Rates of hemorrhage events were reported in five bevacizumab intervention arms (Perez et al., 2009; Piperno-Neumann et al., 2016; McWilliams et al., 2018; Yan et al., 2021). Data were combined using log conversion. The overall rate of hemorrhage events was 6.7% (95% CI, 1.5%–29.1%, *I* ^2^ = 94%, and *p* < 0.01), and the overall rate of grade III/IV hemorrhage events was 0.8% (95% CI, 0%–2.0%, *I* ^2^ = 0%, and *p* = 0.49).

Rates of anemia events were reported in 10 bevacizumab intervention arms (Varker et al., 2007; Del Vecchio et al., 2010; Grignol et al., 2011; Kottschade et al., 2013; Piperno-Neumann et al., 2016; McWilliams et al., 2018; Yan et al., 2021). The original rate was used to determine the overall rate of anemia events: 5.1% (95% CI, 1.7%–8.6%, *I* ^2^ = 60%, and *p* < 0.01). Rates of grade III/IV anemia events were reported in 12 bevacizumab intervention arms (Varker et al., 2007; Del Vecchio et al., 2010; Grignol et al., 2011; Kevin et al., 2012; Von Moos et al., 2012; Kottschade et al., 2013; Piperno-Neumann et al., 2016; McWilliams et al., 2018; Yan et al., 2021). The original rates was used to determine the overall rate of grade III/IV anemia events: 1.6% (95% CI, 0.5%–2.8%, *I* ^2^ = 29%, and *p* = 0.16). Tables 3, 4 show the combined rates of each type of adverse event.

**TABLE 3:** AE rate based on the bevacizumab group.

AE	Studies involved	Event/total	Rate% (95% CI)
Fatigue	18	604/828	71.0 (60.7–81.3)
Nausea	18	408/815	46.6 (36.1–57.1)
Leukopenia	11	257/551	46.2 (28.1–65.4)
Thrombocytopenia	16	339/760	45.4 (32.5–58.3)
Neutropenia	10	242/543	44.9 (26.8–63.1)
Peripheral neuropathy	6	191/423	43.1 (27.0–59.2)
Anaemia	16	370/749	43.1 (25.8–61.2)
Hypertension	21	294/880	32.4 (24.5–40.3)
Diarrhoea	16	174/623	28.8 (19.9–37.7)
Arthralgia	6	93/329	28.0 (23.1–32.8)
Vomit	7	118/414	26.7 (17.1–36.3)
Headache	10	91/454	26.6 (13.4–39.7)
Proteinuria	15	141/530	26.3 (17.3–35.2)
Anorexia	12	124/499	26.2 (17.2–39.9)
Loss of weight	7	54/368	25.5 (6.3–44.7)
Gastritis	11	101/503	23.7 (10.4–40.2)
Myalgia	9	95/403	22.6 (12.3–34.8)
Abdominal pain	8	83/404	20.7 (12.4–29.0)
Epistaxis	12	128/574	20.3 (12.4–28.1)
Dyspnea	12	88/506	19.8 (9.9–29.7)
Sensory nerve abnormality	5	52/277	18.7 (8.1–32.3)
Pyrexia	11	73/462	18.0 (6.1–34.5)
Constipation	12	111/501	17.3 (9.0–27.2)
Allotriogeusia	8	53/395	15.6 (6.7–27.5)
Rash	13	78/520	15.1 (8.1–28.1)
Vertigo	6	38/236	13.8 (9.4–18.1)
Cough	7	45/284	13.3 (9.5–17.2)
Febrile neutropenia	7	23/386	10.1 (0.6–29.0)
Edema	9	32/401	9.9 (2.8–20.8)
Pruritus	5	24/336	7.9 (2.1–29.0)
Allergy	7	34/405	6.9 (4.4–9.3)
Hemorrhage	5	47/306	6.7 (1.5–29.1)
Dyspepsia	7	30/362	6.2 (3.7–8.6)
Dehydration	7	25/345	6.2 (3.5–8.9)
Insomnia	7	27/363	5.6 (3.3–8.0)
Thrombosis	10	27/423	5.1 (1.7–8.6)
Hypokalemia	5	10/192	5.1 (0–11.3)
Bronchopulmonary hemorrhage	5	22/354	4.7 (0–17.7)
Hyponatremia	4	11/186	4.5 (1.6–7.5)
Pulmonary embolism	4	10/224	3.9 (1.3–6.4)
Hypomagnesemia	5	9/329	2.7 (0–12.1)
Dysphagia	6	8/337	1.2 (0–2.3)

**TABLE 4:** III/IV AE rate based on the bevacizumab group.

III/IV AEs	Studies involved	Event/total	Rate % (95% CI)
III/IV neutropenia	15	209/779	19.2 (9.0–32.0)
III/IV leukopenia	10	75/564	13.3 (5.7–20.8)
III/IV peripheral neuropathy	5	38/351	9.3 (2.9–15.8)
III/IV thrombocytopenia	15	54/642	6.2 (3.3–9.2)
III/IV anemia	13	35/557	4.3 (2.0–7.6)
III/IV pulmonary embolism	4	9/224	3.7 (1.2–6.2)
III/IV febrile neutropenia	4	10/232	3.5 (0.9–6.1)
III/IV dyspnea	9	16/352	3.2 (1.4–5.0)
III/IV vomit	7	19/456	2.6 (1.0–4.1)
III/IV thrombosis	12	24/628	1.6 (0.5–2.8)
III/IV allergy	4	5/251	1.0 (0–2.6)
III/IV hemorrhage	5	6/369	0.8 (0–2.0)

### 3.5 Results of meta-analysis based on RCTs

This systematic review included five RCTs in total. For two of the five RCTs, we performed a meta-analysis (Kevin et al., 2012; Yan et al., 2021). The other three RCTs were excluded from the analysis due to insufficient data (U.S.National Library of Medicine, 2018) or because bevacizumab was used in both the experimental and control groups (Varker et al., 2007; McWilliams et al., 2018).

The analysis included 328 patients with malignant melanoma from the two RCTs, with patient age ranging from 27–85 years, including 58.2% men. Both RCTs used paclitaxel + carboplatin as the chemotherapeutic agent, with the experimental group combining the chemotherapeutic agent with bevacizumab and the control group not using bevacizumab. In this section, we examined PFS, OS, ORR, and adverse event rates using data from the two RCTs.

#### 3.5.1 Efficacy

##### 3.5.1.1 PFS

**FIGURE 7:**
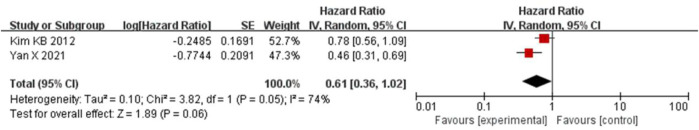
Forest plot comparing PFS in the bevacizumab group with PFS in the chemotherapy group.

The PFS was combined using a random-effects model due to the apparent heterogeneity (*I* ^2^ = 74%, *p* = 0.05). The results showed that bevacizumab in combination with paclitaxel/carboplatin had no significant benefit in reducing the risk of tumor progression compared to paclitaxel/carboplatin chemotherapy regimens (HR = 0.61, 95% CI, 0.36-1.02, and *p* = 0.06) (Figure 7).

##### 3.5.1.2 OS

**FIGURE 8:**
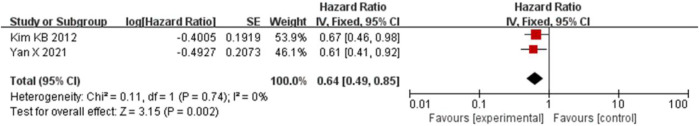
Forest plot comparing OS in the bevacizumab group with OS in the chemotherapy group.

Because the heterogeneity was low (*I* ^2^ = 0% and *p* = 0.74), the OS was combined using a fixed-effects model. Bevacizumab in combination with paclitaxel/carboplatin reduced the risk of patient death compared to chemotherapy administration (HR = 0.64, 95% CI, 0.49-0.85, and *p* < 0.01) (Figure 8).

##### 3.5.1.3 ORR

**FIGURE 9:**
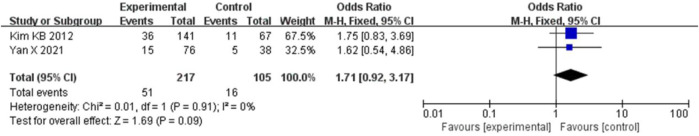
Forest plot comparing ORR in the bevacizumab group with ORR in the chemotherapy group.

The ORR was combined using a fixed-effects model because the heterogeneity was low (*I* ^2^ = 0% and *p* = 0.91). Compared to chemotherapy, bevacizumab in combination with paclitaxel/carboplatin did not significantly increase the ORR (HR = 1.71, 95% CI, 0.92-3.17, and *p* = 0.09) (Figure 9).

#### 3.5.2 Safety

**FIGURE 10:**
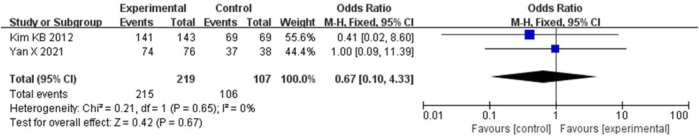
Forest plot comparing the AE rate in the bevacizumab group with the AE rate in chemotherapy.

**FIGURE 11:**
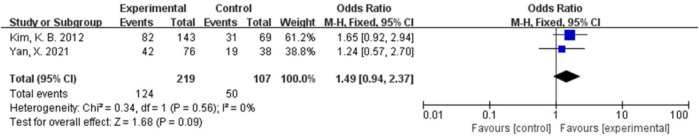
Forest plot comparing the grade III/IV AE rate in the bevacizumab group with the grade III/IV AE rate in chemotherapy.

The AE rate and the grade III/IV AE rate were combined using a fixed-effects model because of relatively low heterogeneity (*I* ^2^ = 0%, *p* = 0.65; *I* ^2^ = 0%, and *p* = 0.56). There were no significant differences in the rates of AEs and III/IV AEs between the two groups (OR = 0.67, 95% CI, 0.10-4.33, and *p* = 0.67 and OR = 1.49, 95% CI, 0.94-2.37, and *p* = 0.09) (Figures 10, 11).

In the analysis of the risk of different kinds of AEs, the results showed that the addition of bevacizumab increased the risk of hypertension in patients (OR = 2.65, 95% CI, 1.06-6.63, and p = 0.04). However, the risk of anemia was lower in the bevacizumab combined with chemotherapy group than in the chemotherapy alone group (OR = 0.58, 95% CI, 0.34–0.99, and *p* = 0.045). Other adverse events (nausea, fatigue, thrombocytopenia, neutropenia, peripheral neuropathy, grade III/IV hypertension, grade III/IV neutropenia, and grade III/IV peripheral neuropathy) were not significantly different between the two groups (Supplementary Tables S3, S4).

### 3.6 Results of subgroup analysis

We divided the bevacizumab intervention arms into subgroups based on different characteristics (combined with chemotherapy, combined with targeted agents, combined with interferon, disease stage, treatment line, study type, and pathologic type) and performed subgroup non-comparative single-arm meta-analyses of ORR, DCR, PFS rate, and OS rate at various time points (3, 6, 9, and 12 months). The results showed that bevacizumab in combination with chemotherapy significantly improved PFS and OS rates at 3 and 6 months. The use of bevacizumab in first-line treatment improved OS rates at 3 and 6 months but had no significant effect on ORR. Patients enrolled in phase III studies had a higher DCR and OS within 12 months and 3-month PFS rate. Other subgroup analyses revealed no statistically significant differences (Supplementary Tables S5–S7).

### 3.7 Sensitivity analysis

Because there was significant heterogeneity in ORR, DCR, PFS rates, and OS rates at 3, 6, 9, and 12 months, we used a one-by-one elimination method to assess the impact of each study on the pooled results to demonstrate stability and sensitivity. The results revealed that all outcomes were reliable and stable (Supplementary Figures S1–S10).

### 3.8 Publication bias

The Begg’s method and the funnel plot method were used to test for publication bias in the ORR of the primary outcome. There was no obvious publication bias (*p* = 0.0768 for Begg’s test). The funnel plot can be seen in Supplementary Material (Supplementary Figure S11).

## 4 Discussion

VEGF is one of the essential growth factors in tumor angiogenesis and is involved in melanoma angiogenesis (Birck et al., 1999). The level of VEGF expression may be significant in independently predicting the OS of melanoma patients (Ugurel et al., 2001). Bevacizumab is a well-known antiangiogenesis medication. By preventing the interaction of VEGF and its receptor and inhibiting the activation of the VEGF signaling pathway, it can reduce angiogenesis and trigger apoptosis of tumor vascular endothelial cells. Simultaneously, it can increase the effect of cytotoxic medications and encourage the creation of an immunosuppressive microenvironment (Ferrara et al., 2004; Rahma and Hodi, 2019). Our study showed that a bevacizumab-based treatment regimen for treating patients with unresectable, stage III or IV malignant melanoma could achieve a better outcome with an ORR of 15.8% and an OS rate of 49.1% at 12 months. According to a real-world retrospective study conducted in China, bevacizumab in combination with carboplatin/paclitaxel has become the most commonly used second-line treatment regimen for adult patients with unresectable, locally advanced, or metastatic melanoma, and the second most commonly used first-line regimen after Endo plus dacarbazine/cisplatin (Cui et al., 2019). Antiangiogenic agents, such as bevacizumab, may be helpful in the treatment of melanoma (Seetharamu et al., 2009). Antiangiogenic drugs combined with paclitaxel/carboplatin regimens are currently recommended by the Chinese Society of Clinical Oncology (CSCO) guidelines as first- or second-line treatment options for patients with metastatic or unresectable stage III or IV cutaneous or acral melanoma with tumor reduction as the primary goal or a large tumor load.

Chemotherapy regimens are routinely employed in the treatment of malignant melanoma. Dacarbazine administered in the first-line treatment of metastatic melanoma promotes tumor remission. Still, it does not improve the survival time of patients (Garbe et al., 2011). The carboplatin/paclitaxel regimen had an ORR and DCR of 13.2% and 34.2%, respectively, with a 12-month OS rate of 36.8% (Rini et al., 2010). Patients receiving paclitaxel monotherapy for malignant melanoma had a stable disease rate of about 29.6%, with no patients achieving objective complete or partial remission and a 12-month overall survival rate of just 30% (Walker et al., 2005). Chemotherapy has traditionally been the major treatment option for patients. However, earlier clinical trials have revealed that chemotherapy has limited efficacy. A subgroup analysis of bevacizumab combined with chemotherapy regimens in this research revealed an ORR of 20.1%, a DCR of 57.1%, and a 12-month OS rate of 51.7%. The combination of bevacizumab with carboplatin/paclitaxel regimens had an ORR of 25.5% and a considerable benefit in prolonging OS and significantly reducing patient mortality risk, which has significant implications for patient survival in oncology. Regarding safety, bevacizumab did not increase patients’ risk of adverse reactions or those that were Grade III or higher. Despite the increase in hypertension cases, bevacizumab did not increase the risk of other safety events. Overall, the results of the addition of bevacizumab to the chemotherapy regimen initially showed that it had good efficacy and tolerable side effects. The combination of carboplatin/paclitaxel and bevacizumab may be an advantageous option for patients who are not eligible for immunotherapy or targeted therapy.

Interferon has immune-stimulating and antiproliferative properties, as well as the ability to inhibit angiogenesis. The combination of interferon and bevacizumab may have a synergistic antiangiogenesis effect. Previous research found that interferon alone had an approximately 15% response rate to malignant melanoma (LEGHA et al., 1997), and bevacizumab alone had no response rate in melanoma patients (Varker et al., 2007). According to this research’s results, interferon plus bevacizumab had an ORR of 16.6% and a 12-month OS rate of 52.3% for malignant melanoma. In the clinical trial included in this study, the combination of bevacizumab and low-dose interferon (1 MU/m2) was less effective, with an ORR of 6.25%, and the two drugs did not enhance antitumor activity. However, the ORR of bevacizumab combined with high-dose interferon (10 MU/m2) reached 24%, suggesting that the combination of bevacizumab and high-dose interferon may be more beneficial in treating melanoma patients. High-dose interferon is commonly used in the clinic as postoperative adjuvant therapy in patients with stage II/III resectable melanoma, but whether it provides clinical benefit in patients with metastatic or unresectable melanoma needs to be validated in larger clinical trials.

BRAF gene mutations are found in approximately 50% of melanoma patients (Cancer Genome Atlas Network, 2015), and RAS, TP53, NF1, and KIT mutations are also common in melanoma patients. Small-molecule targeted drugs improve the prognosis of melanoma patients. The BRAF inhibitor vemurafenib has been approved for patients with unresectable or metastatic melanoma who have BRAF mutations, with a median OS of 13.5 months and an ORR of 52.2% (Si et al., 2018). In addition to vemurafenib, dabrafenib demonstrated efficacy in patients with unresectable melanoma with BRAF mutations, with a median PFS of 5.1 months, significantly longer than that of dacarbazine: 2.1 months (Hauschild et al., 2012). Following clinical trials, we discovered that combining the MEK inhibitor trametinib with the BRAF inhibitor dabrafenib can achieve an ORR of 61% (Si et al., 2020), and a median PFS of 9.4 months, which is significantly longer than that of dabrafenib monotherapy (Flaherty et al., 2012). Imatinib, a KIT inhibitor, can achieve a DCR of 53.5% in acral and mucosal melanoma (Guo et al., 2011). Researchers in clinical trials attempted to treat melanoma by combining various targeted drugs. They tried to combine bevacizumab with small-molecule targeted medications to improve the efficacy of a single drug in treating melanoma. However, subgroup analysis of this study showed that the ORR and DCR of bevacizumab in combination with small-molecule targeted agents were 10.9% and 57.0%, respectively, with a 12-month OS rate of 41.9%. Compared to the results of clinical trials of small-molecule targeted drugs, the addition of bevacizumab does not improve antitumor efficacy. The gene mutation of the patients in the clinical trials included in this study is unknown, which may affect the efficacy results. In the future, larger populations of patients with gene mutations and wild-type genes should be analyzed to determine the more accurate tumor response rate and survival results.

In the 21^st^ century, the use of immune checkpoint inhibitors in melanoma has gradually increased. Several immune checkpoint inhibitors, including the PD-1 inhibitors pembrolizumab, nivolumab, toripalimab, and the CTLA-4 inhibitor ipilimumab, have been approved for melanoma indications and are recommended by melanoma guidelines. Clinical trials have shown that nivolumab and pembrolizumab monotherapy has ORRs ranging from 26% to 44% (Mao et al., 2021). Toripalimab had an ORR of 17.3% in Chinese patients with advanced melanoma (Tang et al., 2020). The combination of nivolumab and ipilimumab further improved OS in melanoma patients, with a median OS of more than 60.0 months and an OS rate at 5 years of 52% (Larkin et al., 2019). Since antiangiogenic drugs can improve the immune microenvironment and facilitate the antitumor effect of immune checkpoint inhibitors, combination therapy with bevacizumab and immune checkpoint inhibitors could be a promising future research direction. In a phase I study, ipilimumab was combined with bevacizumab and had a DCR of 67.4% in advanced melanoma (Hodi et al., 2014). Another multicenter, phase II study in mucosal melanoma found that atezolizumab in combination with bevacizumab had an ORR and DCR of 36.4% and 59.1%, respectively (Si et al., 2021), and based on this study, CSCO recommended atezolizumab in combination with bevacizumab for unresectable or stage IV mucosal melanoma. Although these clinical trials are still ongoing, they have shown that bevacizumab in combination with immune checkpoint inhibitors has an effect. The immuno-antiangiogenic combination therapy may offer new therapeutic hope for melanoma patients. Large, multicenter prospective clinical trials are needed to determine whether this therapy can improve long-term prognosis in patients with advanced melanoma.

This study also discusses the disease stage of the patients included in clinical trials, the treatment lines, the types of studies included in the literature, and the pathological subtype of melanoma. The clinical trials included in this study clearly evaluated the efficacy and adverse events of bevacizumab in the unresectable stage III or IV malignant melanoma population, where the unresectable characteristic of the tumor in stage III patients, a population that no longer has access to surgery, was clarified and thus could be included in clinical trials in the same study as stage IV patients. According to the results, bevacizumab was more beneficial in stage III patients than in stage IV patients. The ORR, DCR, rate of PFS, and rate of OS were all improved in stage III patients compared to stage IV patients, with more apparent benefits in prolonging patient survival. Regarding treatment lines, patients who received bevacizumab as the first-line treatment had higher efficacy, and the OS rate of patients at 3 and 6 months showed clear benefits. Compared to patients with stage IV tumor, the disease in stage III tumor patients was discovered earlier, the tumor advanced modestly, there were no distant metastases, and the condition was easier to manage. Patients who received first-line treatment had advantages over those who received second-line treatment regarding their personal health, tumor load, and sensitivity to antitumor medication. They responded better to medications and frequently had better therapeutic effects. RCTs and non-randomized controlled prospective non-comparative clinical studies were compared among the study-type subgroups, and the results showed no significant differences in outcomes between the two subgroups. This suggests that the quality of the studies we included was generally balanced and that the patients were more uniform at baseline. Some clinical trials included in this study made pathological-type restrictions on malignant melanoma, such as mucosal melanoma and uveal melanoma. We performed a subgroup analysis of melanoma pathological classification, and there was no significant difference in the ORR between subgroups, indicating that bevacizumab could achieve good results in treating various types of melanoma.

This study found that the most common adverse events associated with bevacizumab use in patients with melanoma were fatigue, nausea, leukopenia, thrombocytopenia, and neutropenia, in that order. With a 32.4% incidence, hypertension was the most common bevacizumab-related adverse event. Adverse events such as proteinuria, hemorrhage, and thrombosis were also observed. Among grade III and higher adverse events, myelosuppression toxicity was the most common, such as neutropenia, leukopenia, thrombocytopenia, and anemia. Among the adverse events of grade III and higher, associated with bevacizumab, the incidence of thrombosis was only 1.6%, and the incidence of bleeding was only 0.8%. Overall, the safety of bevacizumab is tolerable, but clinical use should still be evaluated based on the specific adverse events experienced by patients. In practice, it is necessary to keep an eye on both the typical adverse events of bevacizumab and any potential negative effects of the combination therapy.

Twenty clinical studies, five RCTs and 15 non-randomized controlled prospective clinical studies, were included in this study. However, the overall combined results were highly heterogeneous. After subgroup analysis, the heterogeneity was reduced to varying degrees, indicating that different treatment regimens, treatment lines, disease staging, pathological types, and study designs contributed to the heterogeneity. Furthermore, clinical heterogeneity increased the degree of overall heterogeneity in this study, such as tumor patients' quality of survival, the specific interventions they had received, and the duration of follow-up after treatment. As a result, we excluded studies with high heterogeneity one by one for sensitivity analysis, demonstrating that the results of this study were robust.

The limitations of this study are as follows. We did not perform subgroup analysis based on the specific treatment regimen of the bevacizumab combination because of the different interventions included in the study, but only on whether the treatment regimen contained chemotherapeutic agents, targeted agents, or interferons. Because all of the patients in this study had unresectable stage III or IV malignant melanoma, the efficacy of bevacizumab in patients with other stages cannot be confirmed, and the meta-analysis results may not be applicable to patients in other stages. There were few randomized controlled trials for bevacizumab in malignant melanoma, and the clinical studies that could be included in this study were limited. All of the included studies were phase II clinical trials, with the majority of them being non-randomized controlled prospective clinical trials with small sample sizes. The original investigators' errors and biases may have an impact on the results.

## 5 Conclusion

Melanoma is a highly malignant cancer that is aggressive and metastatic. Treatment options for malignant melanoma patients are constantly evolving. Chemotherapy, targeted therapy, and immunotherapy are all commonly used in the real world. In parallel, research on the combination of bevacizumab is being conducted. This study demonstrates that bevacizumab combined with chemotherapy, small-molecule targeted agents, or interferon can be effective and well-tolerated in patients with unresectable stage III or IV malignant melanoma. However, to investigate the anticancer effect of bevacizumab combination regimens and the value of antiangiogenic medications in malignant melanoma, more large-scale randomized controlled trials based on the present clinical research are required to conduct in the future.

## Data Availability

The datasets presented in this study can be found in online repositories. The names of the repository/repositories and accession number(s) can be found in the article/Supplementary Material.
